# Crystal structures of three (trichloromethyl)(carbamoyl)disulfanes

**DOI:** 10.1107/S2056989015015893

**Published:** 2015-09-12

**Authors:** Barbara L. Goldenberg, Victor G. Young Jr, George Barany

**Affiliations:** a Department of Chemistry, University of Minnesota, Minneapolis, MN 55455, USA

**Keywords:** crystal structures, carbamoyl disulfanes, hydrogen bonding, *Z* = 16, *Z*′ = 2, halogen bonding

## Abstract

The present paper reports crystallographic studies on three related compounds that were of inter­est as precursors for synthetic and mechanistic work in organosulfur chemistry, as well as to model nitro­gen-protecting groups: (*N*-methyl­carbamo­yl)(tri­chloro­meth­yl)disulfane, C_3_H_4_Cl_3_NOS_2_, (**1**), (*N*-benzyl­carbamo­yl)(tri­chloro­meth­yl)disulfane, C_9_H_8_Cl_3_NOS_2_, (**2**), and (*N*-methyl-*N*-phenyl­carbamo­yl)(tri­chloro­meth­yl)disulfane, C_9_H_8_Cl_3_NOS_2_, (**3**). Their mol­ecular structures, with similar bond lengths and angles for the CCl_3_SS(C=O)N moieties, are confirmed. Compounds (**1**) and (**3**) both crystallized with two independent mol­ecules in the asymmetric unit. Classical hydrogen bonding, as well as chlorine-dense regions, are evident in the crystal packing for (**1**) and (**2**). In the crystal of (**1**), mol­ecules are linked *via* N—H⋯O hydrogen bonds forming chains along [110], which are linked by short Cl⋯Cl and S⋯O contacts forming sheets parallel to (001). In the crystal of (**2**), mol­ecules are linked *via* N—H⋯O hydrogen bonds forming chains along [001], which in turn are linked by pairs of short O⋯Cl contacts forming ribbons along the *c-*axis direction. In the crystal of (**3**), there are no classical hydrogen bonds present and the chlorine-dense regions observed in (**1**) and (**2**) are lacking.

## Abstract 2

The present paper reports crystallographic studies on three related compounds that were of inter­est as precursors for synthetic and mechanistic work in organosulfur chemistry, as well as to model nitro­gen-protecting groups.

## Chemical context   

**Figure 1:**
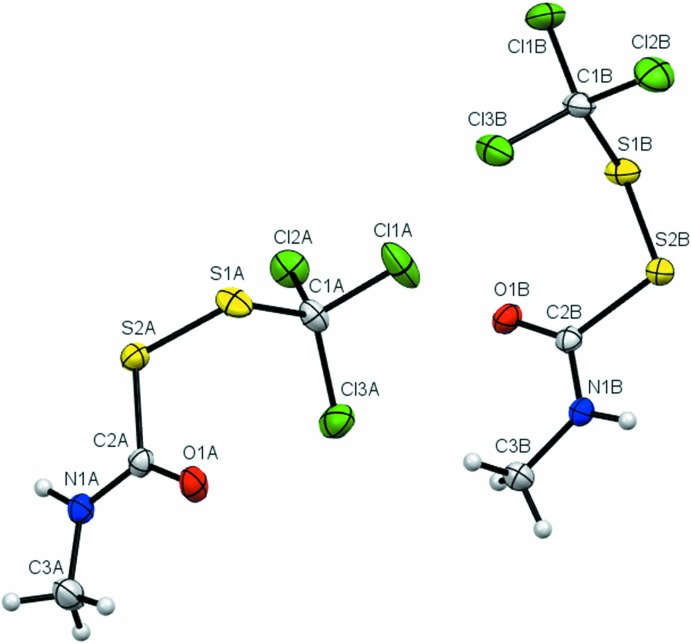
The mol­ecular structure of compound (**1**) showing the atom-labelling scheme, with two mol­ecules (*Z*′ = 2) per asymmetric unit. Displacement ellipsoids are drawn at the 50% probability level.

**Figure 2:**
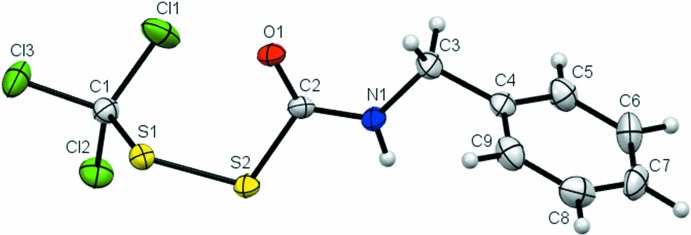
The mol­ecular structure of compound (**2**) showing the atom-labelling scheme. Displacement ellipsoids are drawn at the 50% probability level.

**Figure 3:**
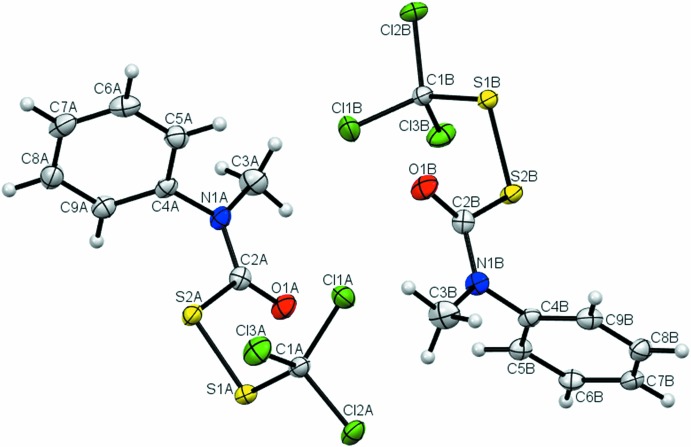
The mol­ecular structure of compound (**3**) showing the atom-labelling scheme, with two mol­ecules (*Z*′ = 2) per asymmetric unit. Displacement ellipsoids are drawn at the 50% probability level.

Carbamoyl disulfanes were first reported by Harris (1960 ▸). This family of compounds has served as useful model compounds for synthetic and mechanistic work in organosulfur chemistry and nitro­gen-protecting-group development (Barany & Merrifield, 1977 ▸; Barany *et al.*, 1983 ▸; Schroll & Barany, 1986 ▸; Barany *et al.*, 2005 ▸; Schrader *et al.*, 2011 ▸). The tri­chloro­methyl derivatives reported here, (tri­chloro­meth­yl)(*N*-methyl­carbamo­yl)disulfane, (**1**) (Fig. 1 ▸), (tri­chloro­meth­yl)(*N*-benz­yl­carbamo­yl)disulfane, (**2**) (Fig. 2 ▸), and (tri­chloro­meth­yl)(*N*-methyl-*N*-phenyl­carbamo­yl)disulfane, (**3**) (Fig. 3 ▸), are partic­ularly stable. All three compounds have been stored under ambient conditions for periods in the range of two to four decades, with no evidence of decomposition based on unchanged ^1^H NMR spectra and melting points.
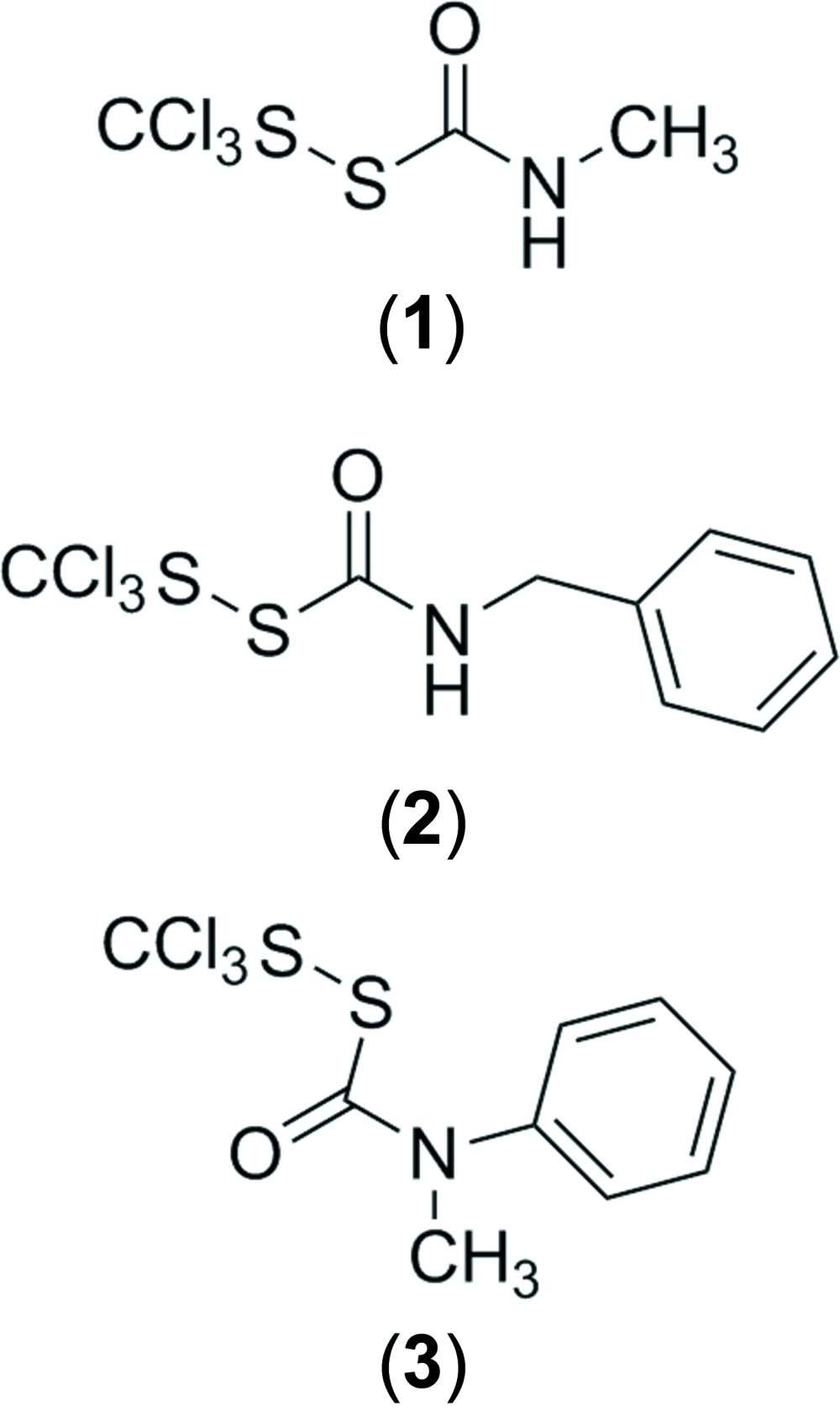


## Structural commentary   

The three (tri­chloro­meth­yl)(carbamo­yl)disulfanes differ in the substituents on the carbamoyl nitro­gen, but the bond lengths and angles of the common CCl_3_SS(C=O)N moieties of each are markedly similar for the two mol­ecules in the asymmetric units of (**1**) and (**3**), as well as for the single conformation of (**2**) (Tables 1 ▸ and 2 ▸). The corresponding structural features of (**3**) are also similar to the bond lengths and torsion angles of other carbamoyl disulfanes that include an SS(C=O)N(Me)Ph chain, including, for example, bis­(*N*-methyl-*N*-phenyl­carbamo­yl)disulfane (ZAQWUL, formula [Ph(Me)N(C=O)S]_2_) (Schroll *et al.*, 2012 ▸) and (*N*-methyl-*N*-phenyl­carbamo­yl)(*N*-methyl-*N*-phenyl­amino)­disulfane [formula Ph(Me)N(C=O)SSN(Me)Ph] (Henley *et al.*, 2015 ▸).

## Supra­molecular features   

**Figure 4:**
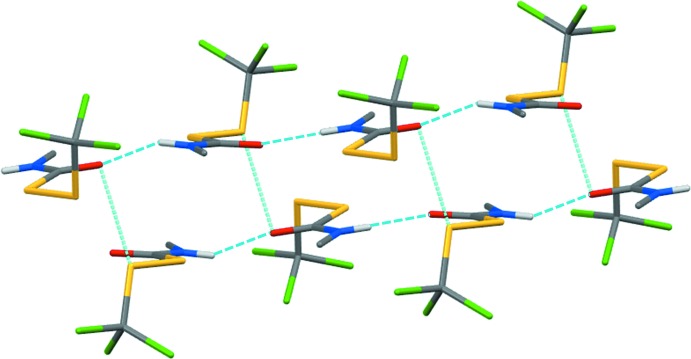
Hydrogen-bonded chains of (**1**) are linked by S1*A*⋯O1*B* contacts. Only H atoms involved in N—H⋯O=C bonds are shown.

**Figure 5:**
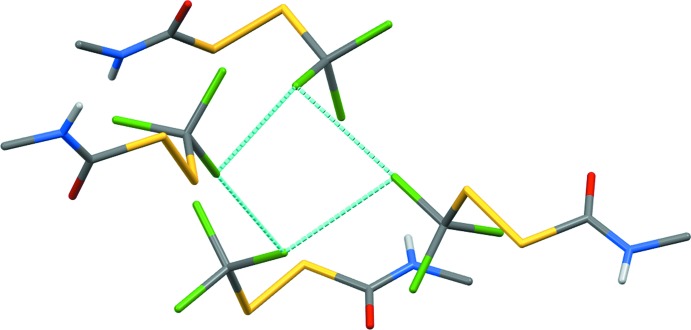
A chlorine from each of four mol­ecules of (**1**), in separate chains, form a short-contact skew quadrilateral. Only H atoms involved in N—H⋯O=C bonds are shown.

The three compounds arrange in three distinct packing configurations. The two nearly superimposable mol­ecular structures of (**1**) are alternately hydrogen-bonded (NH⋯O=C) in chains along [110] (Table 3 ▸). Successive mol­ecules of each of two chains are linked by 3.162 (1) Å S1*A*⋯O1*B* contacts, 0.157 Å less than their van der Waals radii sum (Fig. 4 ▸). Additional packing features result in a *Z* = 16 unit cell. A chlorine from each of four mol­ecules – in separate hydrogen-bonded chains – form a short-contact skew quadrilateral with inter­molecular contact distances of 3.4304 (8) Å (−0.070 Å less than their van der Waals radii sum) and 3.3463 (8) Å (−0.154 Å less than their van der Waals radii sum), Cl3*B*⋯Cl1*A*⋯Cl3*B* and Cl1*A*⋯Cl3*B*⋯Cl1*A* angles 73.40 (2) and 82.01 (2)°, and Cl3*B*⋯Cl1*A*⋯Cl3*B*⋯Cl1*A* and Cl1*A*⋯Cl3*B*⋯Cl1*A*⋯Cl3*B* torsion angles −50.45 (2) and 48.78 (2)°. These result in chlorine-dense regions of the crystal structure (Fig. 5 ▸), and the formation of sheets parallel to (001). Halogen bonding involving tri­chloro­methyl groups in supra­molecular structures was described by Rybarczyk-Pirek *et al.* (2013 ▸).

**Figure 6:**
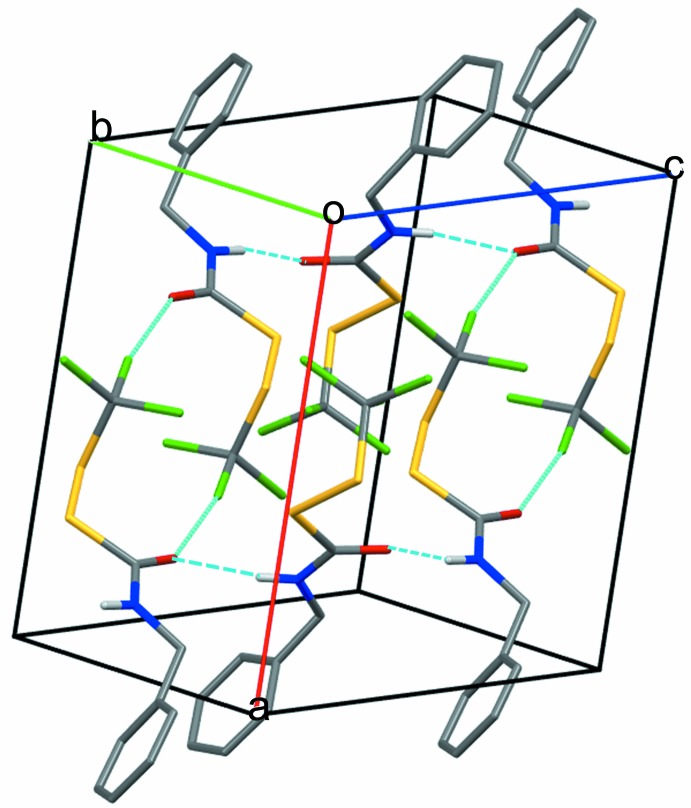
Packing structure of (**2**). Hydrogen-bonded chains are linked by pairs of O1⋯Cl3 contacts. H atoms are not shown unless they participate in hydrogen bonding.

The unit cell of (**2**) consists of pairs of hydrogen-bonded dimers about an inversion center. The mol­ecules in each dimer are linked by NH⋯O=C hydrogen bonds (Table 4 ▸), which extend into hydrogen-bonded mol­ecular chains along [001]. A network of linked chains is formed by O1⋯Cl3 contacts. Two O1⋯Cl3 contacts [3.028 (2) Å, 0.242 Å less than their van der Waals radii sum] form between each pair of mol­ecules in separate hydrogen-bonded chains, and the links extend throughout the chains in alternate mol­ecules. In this way, each hydrogen-bonded chain has extensive links to two other chains. The resulting structure features alternating layers of tri­chloro­methyl and benzyl groups (Fig. 6 ▸).

**Figure 7:**
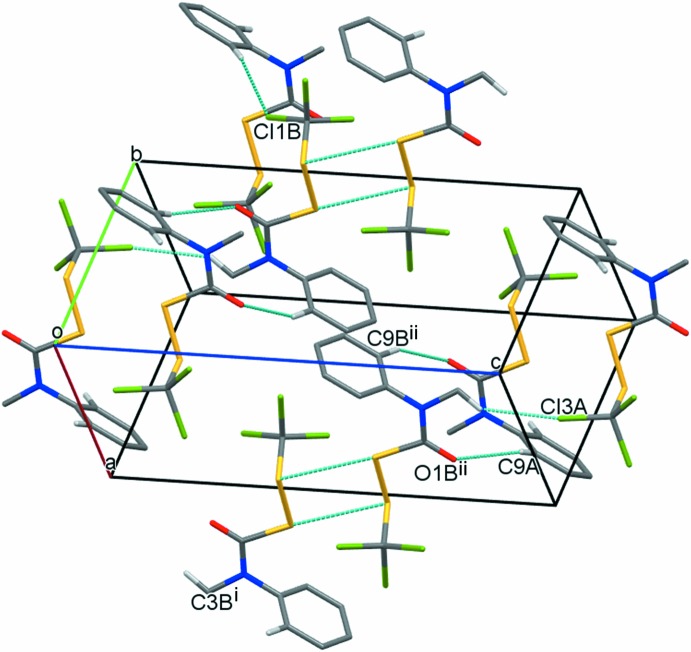
Packing diagram for (**3**). H atoms are not shown unless they participate in hydrogen bonding. [Symmetry codes: (i) −*x* + 1, −*y*, −*z*; (ii) *x*, *y* + 1, *z*.]

Compound (**3**) has no available classical hydrogen bonding and lacks the chlorine-dense regions of (**1**) and (**2**). Of the two conformations available for (**3**), it is noteworthy that the four sulfurs of two adjacent mol­ecules of (**3** ***b***) are positioned in a parallelogram [angles 80.65 (2) and 99.35 (2)°, torsion angle 0.00 (2)°] with inter­molecular contact distances of 3.5969 (8) Å, slightly less than the sum of their van der Waals radii; no such configuration is evident for mol­ecules of (**3** ***a***). Fig. 7 ▸ shows a schematic view of the inter­molecular inter­actions. A pair of non-classical hydrogen bonds [C9*A*—H9*AA*⋯O1*B* and C9*B*—H9B*A*⋯O1*A*, with H⋯C contact distances 2.55 and 2.54 Å, C⋯O distances of 3.360 (3) and 3.432 (3) Å, and C—H⋯O angles of 143 and 157°] connect (**3** ***a***) and (**3** ***b***) mol­ecules. Two additional non-classical hydrogen bonds [C5*A*—H5*AA*⋯Cl1*B* and C3*B*—H3*BA*⋯Cl3*A*, with H⋯Cl contact distances 2.82 and 2.81 Å, C⋯Cl distances of 3.732 (2) and 3.649 (2) Å, and C—H⋯Cl angles of 161 and 144°] are shown.

## Database survey   

Crystal structures for two additional carbamoyl disulfanes have been reported: bis­(indolylcarbamo­yl)disulfane (BOWGAV, formula [C_8_H_6_N(C=O)S]_2_) (Bereman *et al.*, 1983 ▸) and bis­(*N*,*N*-di­cyclo­hexyl­carbamo­yl)disulfane (UDALER, [cHex_2_N(C=O)S]_2_) (Li *et al.*, 2006 ▸). Their mol­ecular structures are consistent with those of the three compounds reported here. Neither of these comparison structures contains halogen atoms or supra­molecular hydrogen bonds. The crystal structure of 1,7-bis­(tri­chloro­meth­yl)hepta­sulfane contains both short Cl⋯Cl contacts and a parallelogram (four sulfurs) formed from the tri­chloro­methyl-adjacent S–S bonds of two mol­ecules (REHKUK; Steudel *et al.*, 1995 ▸).

## Synthesis and crystallization   

**Figure 8:**
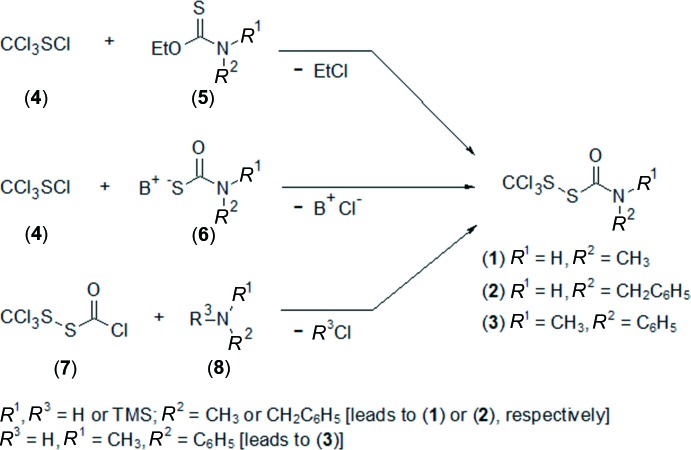
Synthetic routes to (tri­chloro­meth­yl)(carbamo­yl)disulfanes, (**1**), (**2**) and (**3**). See text for further details.

Compounds (**1**) (Harris, 1960 ▸; Barany *et al.*, 2005 ▸), (**2**) (Barany *et al.*, 2005 ▸), and (**3**) (Barany *et al.*, 1983 ▸; Schroll & Barany, 1986 ▸) were synthesized and crystallized as outlined in Fig. 8 ▸ and described in the referenced publications. The reaction of (**4**) plus (**5**), shown in the top pathway of Fig. 8 ▸, is termed the Harris reaction (Harris, 1960 ▸). For the alternative Harris pathway shown in the middle of Fig. 8 ▸, compound (**6**), a thio­carbamate salt, is typically made by reaction of carbonyl sulfide (COS) with a primary or secondary amine HN*R* ^1^ *R* ^2^. Therefore B^+^ is usually the appropriate ammonium counter-ion H_2_N^+^ *R* ^1^ *R* ^2^. Finally, several variations of acyl­ation chemistry are summarized in the bottom pathway of Fig. 8 ▸, as originally worked out by Barany *et al.* (2005 ▸). When *R* ^3^ = H, starting amine HN*R* ^1^ *R* ^2^ is present in sufficient excess so that a second equivalent of amine can absorb the HCl co-product. When *R* ^1^ and/or *R* ^3^ = TMS, stoichiometric ratios can be used, since co-product TMS-Cl is neutral. Note that for some reactions, a TMS group attached to N becomes an H after aqueous workup.

## Refinement   

Crystal data, data collection and structure refinement details are summarized in Table 5 ▸. N—H hydrogen atoms were refined positionally, with restrained *d*(N—H) = 0.85 (1)Å. H atoms attached to C were idealized (C—H: 0.95 Å, C—H_2_: 0.99 Å, C—H_3:_ 0.98 Å). In all cases, *U*_iso_(H) = *x* × *U*_eq_(Host), x = 1.2 except for methyl groups, where *x* = 1.5.

## Supplementary Material

Crystal structure: contains datablock(s) 1, 2, 3. DOI: 10.1107/S2056989015015893/bg2565sup1.cif

Supporting information file. DOI: 10.1107/S2056989015015893/bg25651sup2.cdx

Structure factors: contains datablock(s) 1. DOI: 10.1107/S2056989015015893/bg25651sup8.hkl

Supporting information file. DOI: 10.1107/S2056989015015893/bg25652sup3.cdx

Structure factors: contains datablock(s) 2. DOI: 10.1107/S2056989015015893/bg25652sup9.hkl

Structure factors: contains datablock(s) 3. DOI: 10.1107/S2056989015015893/bg25653sup10.hkl

Supporting information file. DOI: 10.1107/S2056989015015893/bg25653sup4.cdx

Supporting information file. DOI: 10.1107/S2056989015015893/bg25651sup8.cml

Supporting information file. DOI: 10.1107/S2056989015015893/bg25652sup9.cml

Supporting information file. DOI: 10.1107/S2056989015015893/bg25653sup10.cml

CCDC references: 1420526, 1420527, 1420528

Additional supporting information:  crystallographic information; 3D view; checkCIF report

## Figures and Tables

**Table 1:** Selected bond lengths (Å) and angles (°) for CCl_3_SS(C=O)N moieties

	(**1*a***)	(**1*b***)	(**2**)	(**3*a***)	(**3*b***)
S1—C1	1.8242 (18)	1.8261 (18)	1.826 (3)	1.824 (2)	1.822 (2)
S1—S2	2.0100 (7)	2.0126 (6)	2.0099 (11)	2.0202 (7)	2.0160 (7)
S2—C2	1.8367 (17)	1.8426 (17)	1.842 (3)	1.856 (2)	1.842 (2)
O1—C2	1.214 (2)	1.212 (2)	1.213 (4)	1.208 (2)	1.211 (2)
N1—C2	1.322 (2)	1.324 (2)	1.319 (4)	1.345 (3)	1.346 (3)
N1—C3	1.458 (2)	1.460 (2)	1.475 (4)	1.467 (3)	1.460 (3)
N1—C4	–	–	–	1.440 (3)	1.447 (3)
					
C1—S1—S2	103.09 (6)	103.10 (6)	103.68 (11)	102.38 (7)	104.40 (7)
C2—S2—S1	102.20 (6)	101.43 (6)	101.40 (10)	99.96 (7)	101.59 (7)
C2—N1—C3	121.71 (15)	120.35 (14)	121.8 (3)	118.95 (18)	119.49 (18)
O1—C2—N1	126.31 (16)	126.23 (16)	126.4 (3)	125.9 (2)	126.4 (2)
O1—C2—S2	123.02 (13)	122.17 (13)	122.4 (2)	122.09 (16)	122.96 (16)
N1—C2—S2	110.67 (12)	111.58 (12)	111.2 (2)	111.99 (15)	110.65 (14)

**Table 2:** Comparison of selected torsion angles (°)

	(**1*a***)	(**1*b***)	(**2**)	(**3*a***)	(**3*b***)
C1—S1—S2—C2	93.63 (8)	93.49 (8)	96.54 (14)	92.91 (10)	−95.23 (10)
C3—N1—C2—O1	3.3 (3)	1.6 (3)	−1.3 (5)	0.3 (3)	−0.8 (3)
C3—N1—C2—S2	−176.22 (14)	−176.67 (12)	−178.2 (3)	−179.98 (15)	179.73 (16)
S1—S2—C2—O1	2.87 (16)	−0.66 (15)	−2.5 (3)	10.32 (19)	6.32 (19)
S1—S2—C2—N1	−177.64 (11)	177.64 (11)	174.6 (2)	−169.40 (14)	−174.23 (13)
C2—N1—C4—C9	–	–	–	−72.9 (3)	93.8 (2)
C2—N1—C4—C5	–	–	–	109.7 (2)	−86.4 (3)
C3—N1—C4—C9	–	–	–	104.1 (2)	−78.0 (3)
C3—N1—C4—C5	–	–	–	−73.3 (3)	101.8 (2)

**Table 3:** Hydrogen-bond geometry (Å, °) for (**1**)

*D*—H⋯*A*	*D*—H	H⋯*A*	*D*⋯*A*	*D*—H⋯*A*
N1*A*—H1*AA*⋯O1*B* ^i^	0.86 (1)	1.94 (1)	2.7825 (18)	164 (2)
N1*B*—H1*BA*⋯O1*A* ^ii^	0.86 (1)	1.97 (1)	2.8231 (18)	175 (2)

Symmetry codes: (i) 
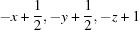
; (ii) 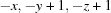.

**Table 4:** Hydrogen-bond geometry (Å, °) for (**2**)

*D*—H⋯*A*	*D*—H	H⋯*A*	*D*⋯*A*	*D*—H⋯*A*
N1—H1*A*⋯O1^i^	0.87 (1)	2.02 (1)	2.887 (3)	174 (3)

Symmetry code: (i) 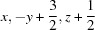.

**Table 5:** Experimental details

	(**1**)	(**2**)	(**3**)
Crystal data			
Chemical formula	C_3_H_4_Cl_3_NOS_2_	C_9_H_8_Cl_3_NOS_2_	C_9_H_8_Cl_3_NOS_2_
*M*_r_	240.54	316.63	316.63
Crystal system, space group	Monoclinic, *C*2/*c*	Monoclinic, *P*2_1_/*c*	Triclinic, *P* 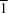
Temperature (K)	123	173	123
*a*, *b*, *c* (Å)	13.1141 (16), 13.9234 (17), 20.172 (3)	11.4247 (17), 13.548 (2), 8.5675 (12)	8.9231 (12), 10.1724 (13), 15.364 (2)
α, β, γ (°)	90, 98.969 (2), 90	90, 103.176 (2), 90	81.964 (2), 81.806 (2), 68.851 (2)
*V* (Å^3^)	3638.3 (8)	1291.2 (3)	1281.5 (3)
*Z*	16	4	4
Radiation type	Mo *K*α	Mo *K*α	Mo *K*α
μ (mm^−1^)	1.40	1.01	1.02
Crystal size (mm)	0.40 × 0.30 × 0.11	0.30 × 0.15 × 0.10	0.25 × 0.20 × 0.09

Data collection			
Diffractometer	Bruker SMART CCD area detector	Bruker SMART CCD area detector	Bruker SMART CCD area detector
Absorption correction	Multi-scan (*SADABS*; Sheldrick, 2008 ▸)	Multi-scan *SADABS*, (Sheldrick, 2008 ▸)	Multi-scan (*SADABS*; Sheldrick, 2008 ▸)
*T*_min_, *T*_max_	0.646, 0.746	0.752, 0.906	0.676, 0.746
No. of measured, independent and observed [*I* > 2σ(*I*)] reflections	21324, 4168, 3556	12180, 2284, 2056	15282, 5790, 4557
*R*_int_	0.030	0.041	0.034
(sin θ/λ)_max_ (Å^−1^)	0.650	0.596	0.649

Refinement			
*R*[*F* ^2^ > 2σ(*F* ^2^)], *wR*(*F* ^2^), *S*	0.025, 0.061, 1.03	0.042, 0.080, 1.00	0.030, 0.073, 0.97
No. of reflections	4168	2284	5790
No. of parameters	189	148	291
No. of restraints	2	1	0
H-atom treatment	H atoms treated by a mixture of independent and constrained refinement	H atoms treated by a mixture of independent and constrained refinement	H atoms treated by a mixture of independent and constrained refinement
Δρ_max_, Δρ_min_ (e Å^−3^)	0.80, −0.63	0.33, −0.27	0.39, −0.27

Computer programs: *SMART* and *SAINT* (Bruker, 2007 ▸), *SHELXL2014* (Sheldrick, 2015 ▸), *SHELXTL* (Sheldrick, 2008 ▸), *Mercury* (Macrae *et al.*, 2008 ▸), *PLATON* (Spek, 2009 ▸) and *ACD/ChemBioDraw* (ACD/Labs, 2014 ▸).

## supplementary crystallographic information

### (1) (N-Methylcarbamoyl)(trichloromethyl)disulfane Crystal data

**Table:**

C_3_H_4_Cl_3_NOS_2_	*D*_x_ = 1.757 Mg m^−^^3^
*M**_r_* = 240.54	Melting point = 352–353 K
Monoclinic, *C*2/*c*	Mo *K*α radiation, λ = 0.71073 Å
*a* = 13.1141 (16) Å	Cell parameters from 2920 reflections
*b* = 13.9234 (17) Å	θ = 2.5–27.5°
*c* = 20.172 (3) Å	µ = 1.40 mm^−^^1^
β = 98.969 (2)°	*T* = 123 K
*V* = 3638.3 (8) Å^3^	Plate, colorless
*Z* = 16	0.40 × 0.30 × 0.11 mm
*F*(000) = 1920	

### (1) (N-Methylcarbamoyl)(trichloromethyl)disulfane Data collection

**Table:**

Bruker SMART CCD area detector diffractometer	3556 reflections with *I* > 2σ(*I*)
Radiation source: sealed tube	*R*_int_ = 0.030
phi and ω scans	θ_max_ = 27.5°, θ_min_ = 2.0°
Absorption correction: multi-scan (*SADABS*; Sheldrick, 2008)	*h* = −16→17
*T*_min_ = 0.646, *T*_max_ = 0.746	*k* = −18→17
21324 measured reflections	*l* = −26→26
4168 independent reflections	

### (1) (N-Methylcarbamoyl)(trichloromethyl)disulfane Refinement

**Table:**

Refinement on *F*^2^	2 restraints
Least-squares matrix: full	Hydrogen site location: mixed
*R*[*F*^2^ > 2σ(*F*^2^)] = 0.025	H atoms treated by a mixture of independent and constrained refinement
*wR*(*F*^2^) = 0.061	*w* = 1/[σ^2^(*F*_o_^2^) + (0.0241*P*)^2^ + 4.8271*P*] where *P* = (*F*_o_^2^ + 2*F*_c_^2^)/3
*S* = 1.03	(Δ/σ)_max_ = 0.002
4168 reflections	Δρ_max_ = 0.80 e Å^−^^3^
189 parameters	Δρ_min_ = −0.63 e Å^−^^3^

### (1) (N-Methylcarbamoyl)(trichloromethyl)disulfane Special details

**Table:**

Experimental. Compound (1) (Harris, 1960; Barany *et al.*, 2005) was synthesized and crystallized as outlined in the Scheme and described in the referenced publications.
Geometry. All e.s.d.'s (except the e.s.d. in the dihedral angle between two l.s. planes) are estimated using the full covariance matrix. The cell e.s.d.'s are taken into account individually in the estimation of e.s.d.'s in distances, angles and torsion angles; correlations between e.s.d.'s in cell parameters are only used when they are defined by crystal symmetry. An approximate (isotropic) treatment of cell e.s.d.'s is used for estimating e.s.d.'s involving l.s. planes.

### (1) (N-Methylcarbamoyl)(trichloromethyl)disulfane Fractional atomic coordinates and isotropic or equivalent isotropic displacement parameters (Å^2^)

**Table:**

	*x*	*y*	*z*	*U*_iso_*/*U*_eq_	
Cl1A	−0.00541 (4)	0.37732 (4)	0.35962 (3)	0.04712 (15)	
Cl2A	0.19373 (3)	0.28755 (4)	0.39682 (2)	0.03390 (11)	
Cl3A	0.08631 (4)	0.35934 (3)	0.49989 (3)	0.03684 (12)	
S1A	−0.00492 (3)	0.19396 (3)	0.41765 (2)	0.02805 (11)	
S2A	0.08261 (4)	0.10871 (3)	0.48401 (2)	0.02537 (10)	
O1A	−0.04393 (10)	0.18404 (9)	0.56382 (7)	0.0302 (3)	
N1A	0.08133 (11)	0.08323 (10)	0.61229 (7)	0.0239 (3)	
H1AA	0.1296 (12)	0.0437 (12)	0.6058 (10)	0.029*	
C1A	0.07085 (13)	0.30414 (13)	0.42009 (9)	0.0255 (4)	
C2A	0.02947 (13)	0.13163 (11)	0.56154 (9)	0.0211 (3)	
C3A	0.05085 (16)	0.08425 (15)	0.67878 (9)	0.0334 (4)	
H3AA	0.0441	0.0181	0.6942	0.050*	
H3AB	−0.0155	0.1174	0.6767	0.050*	
H3AC	0.1034	0.1179	0.7102	0.050*	
Cl1B	0.27558 (4)	0.53778 (4)	0.16143 (2)	0.03787 (12)	
Cl2B	0.08921 (4)	0.64264 (4)	0.17392 (3)	0.04065 (13)	
Cl3B	0.15634 (4)	0.48946 (4)	0.26583 (2)	0.03589 (12)	
S1B	0.28444 (4)	0.66744 (3)	0.27052 (2)	0.02689 (10)	
S2B	0.19567 (4)	0.71821 (3)	0.33553 (2)	0.02639 (10)	
O1B	0.29056 (10)	0.57063 (9)	0.40606 (6)	0.0273 (3)	
N1B	0.18133 (11)	0.65897 (10)	0.45778 (7)	0.0222 (3)	
H1BA	0.1388 (12)	0.7061 (11)	0.4536 (10)	0.027*	
C1B	0.19848 (14)	0.58421 (13)	0.21883 (8)	0.0254 (4)	
C2B	0.22943 (12)	0.63574 (11)	0.40702 (8)	0.0195 (3)	
C3B	0.20171 (14)	0.60575 (13)	0.52084 (9)	0.0262 (4)	
H3BA	0.1735	0.6412	0.5558	0.039*	
H3BB	0.2764	0.5979	0.5341	0.039*	
H3BC	0.1690	0.5424	0.5149	0.039*	

### (1) (N-Methylcarbamoyl)(trichloromethyl)disulfane Atomic displacement parameters (Å^2^)

**Table:**

	*U*^11^	*U*^22^	*U*^33^	*U*^12^	*U*^13^	*U*^23^
Cl1A	0.0370 (3)	0.0591 (3)	0.0472 (3)	0.0132 (2)	0.0127 (2)	0.0337 (3)
Cl2A	0.0227 (2)	0.0413 (3)	0.0390 (3)	−0.00235 (18)	0.00915 (18)	0.0030 (2)
Cl3A	0.0477 (3)	0.0276 (2)	0.0371 (3)	−0.0091 (2)	0.0123 (2)	−0.00656 (19)
S1A	0.0242 (2)	0.0362 (3)	0.0220 (2)	−0.00899 (18)	−0.00211 (17)	0.00193 (18)
S2A	0.0311 (2)	0.0216 (2)	0.0251 (2)	0.00138 (17)	0.00954 (17)	−0.00147 (16)
O1A	0.0276 (7)	0.0306 (7)	0.0346 (7)	0.0138 (5)	0.0115 (5)	0.0094 (6)
N1A	0.0233 (7)	0.0235 (7)	0.0258 (7)	0.0083 (6)	0.0063 (6)	0.0027 (6)
C1A	0.0236 (8)	0.0281 (9)	0.0250 (9)	−0.0008 (7)	0.0044 (7)	0.0071 (7)
C2A	0.0216 (8)	0.0180 (8)	0.0250 (8)	−0.0005 (6)	0.0072 (7)	0.0001 (6)
C3A	0.0377 (11)	0.0366 (10)	0.0276 (10)	0.0080 (9)	0.0101 (8)	0.0080 (8)
Cl1B	0.0494 (3)	0.0412 (3)	0.0250 (2)	0.0009 (2)	0.0121 (2)	−0.00839 (19)
Cl2B	0.0380 (3)	0.0494 (3)	0.0303 (2)	0.0055 (2)	−0.0078 (2)	−0.0003 (2)
Cl3B	0.0457 (3)	0.0362 (3)	0.0246 (2)	−0.0184 (2)	0.00170 (19)	0.00053 (18)
S1B	0.0291 (2)	0.0312 (2)	0.0211 (2)	−0.00846 (18)	0.00602 (17)	−0.00414 (17)
S2B	0.0358 (2)	0.0232 (2)	0.0198 (2)	0.00546 (18)	0.00314 (17)	−0.00014 (16)
O1B	0.0289 (6)	0.0262 (6)	0.0270 (6)	0.0102 (5)	0.0051 (5)	−0.0037 (5)
N1B	0.0237 (7)	0.0207 (7)	0.0227 (7)	0.0074 (6)	0.0047 (6)	−0.0002 (6)
C1B	0.0307 (9)	0.0288 (9)	0.0163 (8)	−0.0028 (7)	0.0023 (7)	−0.0016 (7)
C2B	0.0197 (8)	0.0184 (8)	0.0193 (8)	0.0001 (6)	−0.0003 (6)	−0.0023 (6)
C3B	0.0308 (9)	0.0258 (9)	0.0228 (9)	0.0012 (7)	0.0065 (7)	0.0021 (7)

### (1) (N-Methylcarbamoyl)(trichloromethyl)disulfane Geometric parameters (Å, º)

**Table:**

Cl1A—C1A	1.7736 (18)	Cl1B—C1B	1.7736 (18)
Cl2A—C1A	1.7625 (18)	Cl2B—C1B	1.7696 (19)
Cl3A—C1A	1.7667 (19)	Cl3B—C1B	1.7629 (18)
S1A—C1A	1.8242 (18)	S1B—C1B	1.8261 (18)
S1A—S2A	2.0100 (7)	S1B—S2B	2.0126 (6)
S2A—C2A	1.8367 (17)	S2B—C2B	1.8426 (17)
O1A—C2A	1.214 (2)	O1B—C2B	1.212 (2)
N1A—C2A	1.322 (2)	N1B—C2B	1.324 (2)
N1A—C3A	1.458 (2)	N1B—C3B	1.460 (2)
N1A—H1AA	0.864 (9)	N1B—H1BA	0.857 (9)
C3A—H3AA	0.9800	C3B—H3BA	0.9800
C3A—H3AB	0.9800	C3B—H3BB	0.9800
C3A—H3AC	0.9800	C3B—H3BC	0.9800
			
C1A—S1A—S2A	103.09 (6)	C1B—S1B—S2B	103.10 (6)
C2A—S2A—S1A	102.20 (6)	C2B—S2B—S1B	101.43 (6)
C2A—N1A—C3A	121.71 (15)	C2B—N1B—C3B	120.35 (14)
C2A—N1A—H1AA	120.5 (14)	C2B—N1B—H1BA	119.5 (14)
C3A—N1A—H1AA	117.3 (14)	C3B—N1B—H1BA	120.2 (14)
Cl2A—C1A—Cl3A	108.65 (10)	Cl3B—C1B—Cl2B	108.82 (10)
Cl2A—C1A—Cl1A	109.45 (9)	Cl3B—C1B—Cl1B	109.68 (10)
Cl3A—C1A—Cl1A	110.43 (10)	Cl2B—C1B—Cl1B	109.38 (9)
Cl2A—C1A—S1A	113.54 (10)	Cl3B—C1B—S1B	112.65 (9)
Cl3A—C1A—S1A	112.03 (9)	Cl2B—C1B—S1B	112.28 (10)
Cl1A—C1A—S1A	102.60 (9)	Cl1B—C1B—S1B	103.90 (9)
O1A—C2A—N1A	126.31 (16)	O1B—C2B—N1B	126.23 (16)
O1A—C2A—S2A	123.02 (13)	O1B—C2B—S2B	122.17 (13)
N1A—C2A—S2A	110.67 (12)	N1B—C2B—S2B	111.58 (12)
N1A—C3A—H3AA	109.5	N1B—C3B—H3BA	109.5
N1A—C3A—H3AB	109.5	N1B—C3B—H3BB	109.5
H3AA—C3A—H3AB	109.5	H3BA—C3B—H3BB	109.5
N1A—C3A—H3AC	109.5	N1B—C3B—H3BC	109.5
H3AA—C3A—H3AC	109.5	H3BA—C3B—H3BC	109.5
H3AB—C3A—H3AC	109.5	H3BB—C3B—H3BC	109.5
			
C1A—S1A—S2A—C2A	93.63 (8)	C1B—S1B—S2B—C2B	93.49 (8)
S2A—S1A—C1A—Cl2A	60.37 (10)	S2B—S1B—C1B—Cl3B	−60.94 (10)
S2A—S1A—C1A—Cl3A	−63.18 (10)	S2B—S1B—C1B—Cl2B	62.34 (9)
S2A—S1A—C1A—Cl1A	178.38 (6)	S2B—S1B—C1B—Cl1B	−179.58 (6)
C3A—N1A—C2A—O1A	3.3 (3)	C3B—N1B—C2B—O1B	1.6 (3)
C3A—N1A—C2A—S2A	−176.22 (14)	C3B—N1B—C2B—S2B	−176.67 (12)
S1A—S2A—C2A—O1A	2.87 (16)	S1B—S2B—C2B—O1B	−0.66 (15)
S1A—S2A—C2A—N1A	−177.64 (11)	S1B—S2B—C2B—N1B	177.64 (11)

### (1) (N-Methylcarbamoyl)(trichloromethyl)disulfane Hydrogen-bond geometry (Å, º)

**Table:**

*D*—H···*A*	*D*—H	H···*A*	*D*···*A*	*D*—H···*A*
N1*A*—H1*AA*···O1*B*^i^	0.86 (1)	1.94 (1)	2.7825 (18)	164 (2)
N1*B*—H1*BA*···O1*A*^ii^	0.86 (1)	1.97 (1)	2.8231 (18)	175 (2)

Symmetry codes: (i) −*x*+1/2, −*y*+1/2, −*z*+1; (ii) −*x*, −*y*+1, −*z*+1.

### (2) (N-Benzylcarbamoyl)(trichloromethyl)disulfane Crystal data

**Table:**

C_9_H_8_Cl_3_NOS_2_	*D*_x_ = 1.629 Mg m^−^^3^
*M**_r_* = 316.63	Melting point = 357–359 K
Monoclinic, *P*2_1_/*c*	Mo *K*α radiation, λ = 0.71073 Å
*a* = 11.4247 (17) Å	Cell parameters from 2312 reflections
*b* = 13.548 (2) Å	θ = 2.4–24.9°
*c* = 8.5675 (12) Å	µ = 1.01 mm^−^^1^
β = 103.176 (2)°	*T* = 173 K
*V* = 1291.2 (3) Å^3^	Rod, white
*Z* = 4	0.30 × 0.15 × 0.10 mm
*F*(000) = 640	

### (2) (N-Benzylcarbamoyl)(trichloromethyl)disulfane Data collection

**Table:**

Bruker SMART CCD area detector diffractometer	2056 reflections with *I* > 2σ(*I*)
Radiation source: sealed tube	*R*_int_ = 0.041
phi and ω scans	θ_max_ = 25.1°, θ_min_ = 1.8°
Absorption correction: multi-scan *SADABS*, (Sheldrick, 2008)	*h* = −13→13
*T*_min_ = 0.752, *T*_max_ = 0.906	*k* = −16→16
12180 measured reflections	*l* = −10→10
2284 independent reflections	

### (2) (N-Benzylcarbamoyl)(trichloromethyl)disulfane Refinement

**Table:**

Refinement on *F*^2^	1 restraint
Least-squares matrix: full	Hydrogen site location: mixed
*R*[*F*^2^ > 2σ(*F*^2^)] = 0.042	H atoms treated by a mixture of independent and constrained refinement
*wR*(*F*^2^) = 0.080	*w* = 1/[σ^2^(*F*_o_^2^) + (0.0157*P*)^2^ + 3.520*P*] where *P* = (*F*_o_^2^ + 2*F*_c_^2^)/3
*S* = 1.00	(Δ/σ)_max_ < 0.001
2284 reflections	Δρ_max_ = 0.33 e Å^−^^3^
148 parameters	Δρ_min_ = −0.27 e Å^−^^3^

### (2) (N-Benzylcarbamoyl)(trichloromethyl)disulfane Special details

**Table:**

Experimental. Compound (2) (Barany *et al.*, 2005) was synthesized and crystallized as outlined in the Scheme and described in the referenced publication.
Geometry. All e.s.d.'s (except the e.s.d. in the dihedral angle between two l.s. planes) are estimated using the full covariance matrix. The cell e.s.d.'s are taken into account individually in the estimation of e.s.d.'s in distances, angles and torsion angles; correlations between e.s.d.'s in cell parameters are only used when they are defined by crystal symmetry. An approximate (isotropic) treatment of cell e.s.d.'s is used for estimating e.s.d.'s involving l.s. planes.

### (2) (N-Benzylcarbamoyl)(trichloromethyl)disulfane Fractional atomic coordinates and isotropic or equivalent isotropic displacement parameters (Å^2^)

**Table:**

	*x*	*y*	*z*	*U*_iso_*/*U*_eq_	
Cl1	0.57223 (8)	0.68834 (7)	0.12990 (11)	0.0399 (2)	
Cl2	0.69283 (7)	0.60896 (7)	0.43579 (10)	0.0320 (2)	
Cl3	0.64826 (8)	0.48462 (7)	0.15317 (11)	0.0385 (2)	
S1	0.44925 (7)	0.53104 (6)	0.27940 (9)	0.02193 (18)	
S2	0.37502 (7)	0.64387 (6)	0.37478 (9)	0.0249 (2)	
O1	0.26774 (19)	0.66377 (16)	0.0620 (2)	0.0250 (5)	
N1	0.2069 (2)	0.7676 (2)	0.2344 (3)	0.0247 (6)	
H1A	0.222 (3)	0.785 (2)	0.3348 (16)	0.030*	
C1	0.5911 (3)	0.5817 (2)	0.2514 (4)	0.0249 (7)	
C2	0.2734 (3)	0.6962 (2)	0.1955 (3)	0.0207 (7)	
C3	0.1156 (3)	0.8196 (3)	0.1127 (4)	0.0327 (8)	
H2A	0.0778	0.7728	0.0272	0.039*	
H2B	0.1544	0.8728	0.0632	0.039*	
C4	0.0210 (3)	0.8631 (2)	0.1889 (4)	0.0258 (7)	
C5	0.0178 (3)	0.9637 (3)	0.2167 (4)	0.0319 (8)	
H5A	0.0737	1.0060	0.1833	0.038*	
C6	−0.0662 (3)	1.0032 (3)	0.2928 (5)	0.0374 (9)	
H6A	−0.0681	1.0724	0.3102	0.045*	
C7	−0.1466 (3)	0.9429 (3)	0.3430 (4)	0.0347 (9)	
H7A	−0.2029	0.9701	0.3974	0.042*	
C8	−0.1457 (3)	0.8434 (3)	0.3145 (4)	0.0377 (9)	
H8A	−0.2023	0.8019	0.3481	0.045*	
C9	−0.0627 (3)	0.8028 (3)	0.2371 (4)	0.0333 (8)	
H9A	−0.0632	0.7338	0.2171	0.040*	

### (2) (N-Benzylcarbamoyl)(trichloromethyl)disulfane Atomic displacement parameters (Å^2^)

**Table:**

	*U*^11^	*U*^22^	*U*^33^	*U*^12^	*U*^13^	*U*^23^
Cl1	0.0348 (5)	0.0426 (5)	0.0407 (5)	−0.0039 (4)	0.0055 (4)	0.0208 (4)
Cl2	0.0283 (4)	0.0376 (5)	0.0262 (4)	−0.0033 (4)	−0.0021 (3)	−0.0023 (4)
Cl3	0.0296 (5)	0.0506 (6)	0.0360 (5)	0.0088 (4)	0.0088 (4)	−0.0132 (4)
S1	0.0225 (4)	0.0209 (4)	0.0225 (4)	0.0011 (3)	0.0054 (3)	0.0005 (3)
S2	0.0263 (4)	0.0321 (5)	0.0159 (4)	0.0072 (4)	0.0036 (3)	−0.0006 (3)
O1	0.0283 (12)	0.0321 (13)	0.0143 (11)	0.0036 (10)	0.0043 (9)	−0.0007 (9)
N1	0.0275 (15)	0.0317 (15)	0.0137 (13)	0.0101 (12)	0.0020 (11)	−0.0024 (11)
C1	0.0216 (16)	0.0311 (18)	0.0216 (16)	0.0023 (14)	0.0038 (13)	0.0012 (14)
C2	0.0210 (16)	0.0242 (17)	0.0176 (16)	−0.0015 (13)	0.0059 (12)	0.0018 (13)
C3	0.0319 (19)	0.041 (2)	0.0228 (17)	0.0146 (17)	0.0023 (14)	0.0023 (16)
C4	0.0239 (17)	0.0323 (19)	0.0188 (16)	0.0067 (14)	−0.0004 (13)	0.0013 (14)
C5	0.0242 (18)	0.0318 (19)	0.038 (2)	0.0007 (15)	0.0032 (15)	0.0032 (16)
C6	0.030 (2)	0.0291 (19)	0.052 (2)	0.0074 (16)	0.0059 (17)	−0.0070 (17)
C7	0.0199 (17)	0.048 (2)	0.035 (2)	0.0112 (16)	0.0039 (15)	−0.0053 (17)
C8	0.0246 (18)	0.051 (2)	0.037 (2)	−0.0034 (17)	0.0051 (16)	0.0099 (18)
C9	0.0334 (19)	0.0282 (19)	0.0344 (19)	0.0018 (16)	−0.0006 (16)	−0.0005 (15)

### (2) (N-Benzylcarbamoyl)(trichloromethyl)disulfane Geometric parameters (Å, º)

**Table:**

Cl1—C1	1.764 (3)	C3—H2B	0.9900
Cl2—C1	1.773 (3)	C4—C5	1.386 (5)
Cl3—C1	1.766 (3)	C4—C9	1.391 (5)
S1—C1	1.826 (3)	C5—C6	1.385 (5)
S1—S2	2.0099 (11)	C5—H5A	0.9500
S2—C2	1.842 (3)	C6—C7	1.369 (5)
O1—C2	1.213 (4)	C6—H6A	0.9500
N1—C2	1.319 (4)	C7—C8	1.370 (5)
N1—C3	1.475 (4)	C7—H7A	0.9500
N1—H1A	0.870 (10)	C8—C9	1.389 (5)
C3—C4	1.504 (4)	C8—H8A	0.9500
C3—H2A	0.9900	C9—H9A	0.9500
			
C1—S1—S2	103.68 (11)	H2A—C3—H2B	108.2
C2—S2—S1	101.40 (10)	C5—C4—C9	118.7 (3)
C2—N1—C3	121.8 (3)	C5—C4—C3	120.7 (3)
C2—N1—H1A	117 (2)	C9—C4—C3	120.6 (3)
C3—N1—H1A	121 (2)	C6—C5—C4	120.6 (3)
Cl1—C1—Cl3	109.72 (17)	C6—C5—H5A	119.7
Cl1—C1—Cl2	108.81 (18)	C4—C5—H5A	119.7
Cl3—C1—Cl2	109.94 (17)	C7—C6—C5	120.3 (3)
Cl1—C1—S1	113.13 (17)	C7—C6—H6A	119.9
Cl3—C1—S1	102.59 (17)	C5—C6—H6A	119.9
Cl2—C1—S1	112.48 (17)	C6—C7—C8	119.9 (3)
O1—C2—N1	126.4 (3)	C6—C7—H7A	120.1
O1—C2—S2	122.4 (2)	C8—C7—H7A	120.1
N1—C2—S2	111.2 (2)	C7—C8—C9	120.6 (3)
N1—C3—C4	110.0 (3)	C7—C8—H8A	119.7
N1—C3—H2A	109.7	C9—C8—H8A	119.7
C4—C3—H2A	109.7	C8—C9—C4	119.9 (3)
N1—C3—H2B	109.7	C8—C9—H9A	120.0
C4—C3—H2B	109.7	C4—C9—H9A	120.0
			
C1—S1—S2—C2	96.54 (14)	N1—C3—C4—C9	−72.6 (4)
S2—S1—C1—Cl1	−57.38 (18)	C9—C4—C5—C6	0.8 (5)
S2—S1—C1—Cl3	−175.51 (11)	C3—C4—C5—C6	−177.4 (3)
S2—S1—C1—Cl2	66.42 (17)	C4—C5—C6—C7	0.7 (5)
C3—N1—C2—O1	−1.3 (5)	C5—C6—C7—C8	−1.5 (5)
C3—N1—C2—S2	−178.2 (3)	C6—C7—C8—C9	0.9 (5)
S1—S2—C2—O1	−2.5 (3)	C7—C8—C9—C4	0.5 (5)
S1—S2—C2—N1	174.6 (2)	C5—C4—C9—C8	−1.4 (5)
C2—N1—C3—C4	155.8 (3)	C3—C4—C9—C8	176.8 (3)
N1—C3—C4—C5	105.5 (4)		

### (2) (N-Benzylcarbamoyl)(trichloromethyl)disulfane Hydrogen-bond geometry (Å, º)

**Table:**

*D*—H···*A*	*D*—H	H···*A*	*D*···*A*	*D*—H···*A*
N1—H1*A*···O1^i^	0.87 (1)	2.02 (1)	2.887 (3)	174 (3)

Symmetry code: (i) *x*, −*y*+3/2, *z*+1/2.

### (3) (N-Methyl-N-phenylcarbamoyl)(trichloromethyl)disulfane Crystal data

**Table:**

C_9_H_8_Cl_3_NOS_2_	*F*(000) = 640
*M**_r_* = 316.63	*D*_x_ = 1.641 Mg m^−^^3^
Triclinic, *P*1	Melting point = 327–328 K
*a* = 8.9231 (12) Å	Mo *K*α radiation, λ = 0.71073 Å
*b* = 10.1724 (13) Å	Cell parameters from 2932 reflections
*c* = 15.364 (2) Å	θ = 2.5–27.4°
α = 81.964 (2)°	µ = 1.02 mm^−^^1^
β = 81.806 (2)°	*T* = 123 K
γ = 68.851 (2)°	Plate, colourless
*V* = 1281.5 (3) Å^3^	0.25 × 0.20 × 0.09 mm
*Z* = 4	

### (3) (N-Methyl-N-phenylcarbamoyl)(trichloromethyl)disulfane Data collection

**Table:**

Bruker SMART CCD area detector diffractometer	4557 reflections with *I* > 2σ(*I*)
Radiation source: sealed tube	*R*_int_ = 0.034
phi and ω scans	θ_max_ = 27.5°, θ_min_ = 1.4°
Absorption correction: multi-scan (*SADABS*; Sheldrick, 2008)	*h* = −11→11
*T*_min_ = 0.676, *T*_max_ = 0.746	*k* = −13→12
15282 measured reflections	*l* = −19→19
5790 independent reflections	

### (3) (N-Methyl-N-phenylcarbamoyl)(trichloromethyl)disulfane Refinement

**Table:**

Refinement on *F*^2^	0 restraints
Least-squares matrix: full	Hydrogen site location: inferred from neighbouring sites
*R*[*F*^2^ > 2σ(*F*^2^)] = 0.030	H atoms treated by a mixture of independent and constrained refinement
*wR*(*F*^2^) = 0.073	*w* = 1/[σ^2^(*F*_o_^2^) + (0.0294*P*)^2^ + 0.677*P*] where *P* = (*F*_o_^2^ + 2*F*_c_^2^)/3
*S* = 0.97	(Δ/σ)_max_ = 0.001
5790 reflections	Δρ_max_ = 0.39 e Å^−^^3^
291 parameters	Δρ_min_ = −0.27 e Å^−^^3^

### (3) (N-Methyl-N-phenylcarbamoyl)(trichloromethyl)disulfane Special details

**Table:**

Experimental. Compound (3) (Barany *et al.*, 1983; Schroll & Barany, 1986) was synthesized and crystallized as outlined in the Scheme and described in the reference publications.
Geometry. All e.s.d.'s (except the e.s.d. in the dihedral angle between two l.s. planes) are estimated using the full covariance matrix. The cell e.s.d.'s are taken into account individually in the estimation of e.s.d.'s in distances, angles and torsion angles; correlations between e.s.d.'s in cell parameters are only used when they are defined by crystal symmetry. An approximate (isotropic) treatment of cell e.s.d.'s is used for estimating e.s.d.'s involving l.s. planes.

### (3) (N-Methyl-N-phenylcarbamoyl)(trichloromethyl)disulfane Fractional atomic coordinates and isotropic or equivalent isotropic displacement parameters (Å^2^)

**Table:**

	*x*	*y*	*z*	*U*_iso_*/*U*_eq_	
Cl1A	0.39735 (6)	0.18646 (6)	0.21843 (4)	0.02947 (13)	
Cl2A	0.74662 (6)	0.06953 (6)	0.18508 (4)	0.02861 (13)	
Cl3A	0.54721 (7)	0.15353 (7)	0.04057 (4)	0.03453 (14)	
S1A	0.59372 (6)	0.36579 (6)	0.14069 (4)	0.02254 (12)	
S2A	0.37780 (6)	0.50367 (6)	0.10688 (3)	0.02229 (12)	
O1A	0.34261 (18)	0.50799 (16)	0.28397 (10)	0.0278 (3)	
N1A	0.1137 (2)	0.61782 (18)	0.21527 (11)	0.0216 (4)	
C1A	0.5668 (2)	0.1953 (2)	0.14591 (14)	0.0222 (4)	
C2A	0.2724 (3)	0.5449 (2)	0.21823 (14)	0.0216 (4)	
C3A	0.0124 (3)	0.6590 (3)	0.29789 (15)	0.0330 (5)	
H3AA	0.0804	0.6312	0.3468	0.050*	
H3AB	−0.0674	0.6115	0.3089	0.050*	
H3AC	−0.0433	0.7618	0.2931	0.050*	
C4A	0.0386 (2)	0.6638 (2)	0.13409 (13)	0.0202 (4)	
C5A	−0.0647 (3)	0.6002 (2)	0.11406 (15)	0.0264 (5)	
H5AA	−0.0818	0.5242	0.1525	0.032*	
C6A	−0.1425 (3)	0.6478 (3)	0.03785 (16)	0.0300 (5)	
H6AA	−0.2142	0.6052	0.0244	0.036*	
C7A	−0.1160 (3)	0.7568 (3)	−0.01833 (15)	0.0307 (5)	
H7AA	−0.1693	0.7889	−0.0707	0.037*	
C8A	−0.0124 (3)	0.8198 (2)	0.00094 (15)	0.0301 (5)	
H8AA	0.0066	0.8940	−0.0386	0.036*	
C9A	0.0643 (3)	0.7748 (2)	0.07821 (14)	0.0255 (5)	
H9AA	0.1332	0.8195	0.0924	0.031*	
Cl1B	−0.02011 (7)	0.26039 (6)	0.25446 (4)	0.03331 (14)	
Cl2B	−0.30856 (6)	0.26958 (5)	0.37202 (4)	0.02562 (12)	
Cl3B	−0.02396 (7)	0.27511 (6)	0.43942 (4)	0.03100 (14)	
S1B	−0.04426 (6)	0.01403 (5)	0.38013 (3)	0.02037 (12)	
S2B	0.19477 (6)	−0.06161 (6)	0.39221 (3)	0.02144 (12)	
O1B	0.19752 (18)	−0.07526 (17)	0.21777 (10)	0.0290 (4)	
N1B	0.4406 (2)	−0.17006 (18)	0.27509 (11)	0.0220 (4)	
C1B	−0.0950 (2)	0.2053 (2)	0.36108 (14)	0.0213 (4)	
C2B	0.2795 (3)	−0.1046 (2)	0.27890 (13)	0.0205 (4)	
C3B	0.5317 (3)	−0.2140 (3)	0.19095 (15)	0.0352 (6)	
H3BA	0.4622	−0.1715	0.1431	0.053*	
H3BB	0.5691	−0.3174	0.1926	0.053*	
H3BC	0.6250	−0.1826	0.1807	0.053*	
C4B	0.5250 (2)	−0.2155 (2)	0.35376 (13)	0.0191 (4)	
C5B	0.5752 (2)	−0.1230 (2)	0.39013 (14)	0.0213 (4)	
H5BA	0.5574	−0.0298	0.3626	0.026*	
C6B	0.6515 (3)	−0.1679 (2)	0.46693 (14)	0.0241 (5)	
H6BA	0.6852	−0.1048	0.4926	0.029*	
C7B	0.6789 (2)	−0.3044 (2)	0.50636 (15)	0.0243 (5)	
H7BA	0.7295	−0.3344	0.5596	0.029*	
C8B	0.6322 (3)	−0.3973 (2)	0.46799 (15)	0.0269 (5)	
H8BA	0.6535	−0.4917	0.4943	0.032*	
C9B	0.5547 (2)	−0.3531 (2)	0.39146 (15)	0.0245 (5)	
H9BA	0.5223	−0.4166	0.3652	0.029*	

### (3) (N-Methyl-N-phenylcarbamoyl)(trichloromethyl)disulfane Atomic displacement parameters (Å^2^)

**Table:**

	*U*^11^	*U*^22^	*U*^33^	*U*^12^	*U*^13^	*U*^23^
Cl1A	0.0243 (3)	0.0276 (3)	0.0345 (3)	−0.0101 (2)	0.0015 (2)	0.0022 (2)
Cl2A	0.0235 (3)	0.0256 (3)	0.0300 (3)	0.0005 (2)	−0.0074 (2)	−0.0007 (2)
Cl3A	0.0382 (3)	0.0403 (3)	0.0264 (3)	−0.0111 (3)	−0.0104 (2)	−0.0075 (2)
S1A	0.0169 (3)	0.0241 (3)	0.0257 (3)	−0.0062 (2)	−0.0052 (2)	0.0014 (2)
S2A	0.0195 (3)	0.0243 (3)	0.0189 (3)	−0.0028 (2)	−0.0042 (2)	0.0009 (2)
O1A	0.0284 (8)	0.0337 (9)	0.0205 (8)	−0.0074 (7)	−0.0086 (7)	−0.0031 (7)
N1A	0.0222 (9)	0.0225 (9)	0.0173 (9)	−0.0035 (7)	−0.0029 (7)	−0.0030 (7)
C1A	0.0182 (10)	0.0252 (11)	0.0205 (11)	−0.0041 (9)	−0.0028 (8)	−0.0019 (9)
C2A	0.0244 (11)	0.0194 (10)	0.0215 (11)	−0.0072 (9)	−0.0038 (9)	−0.0026 (8)
C3A	0.0304 (13)	0.0390 (14)	0.0248 (12)	−0.0056 (11)	0.0022 (10)	−0.0100 (10)
C4A	0.0167 (10)	0.0203 (10)	0.0189 (10)	0.0003 (8)	−0.0013 (8)	−0.0054 (8)
C5A	0.0239 (11)	0.0248 (11)	0.0301 (12)	−0.0082 (9)	−0.0023 (9)	−0.0028 (9)
C6A	0.0214 (11)	0.0370 (13)	0.0329 (13)	−0.0081 (10)	−0.0035 (10)	−0.0122 (11)
C7A	0.0214 (11)	0.0432 (14)	0.0200 (11)	0.0001 (10)	−0.0034 (9)	−0.0080 (10)
C8A	0.0284 (12)	0.0304 (12)	0.0255 (12)	−0.0044 (10)	−0.0024 (10)	0.0014 (10)
C9A	0.0238 (11)	0.0241 (11)	0.0273 (12)	−0.0062 (9)	−0.0035 (9)	−0.0028 (9)
Cl1B	0.0299 (3)	0.0319 (3)	0.0323 (3)	−0.0101 (2)	0.0021 (2)	0.0089 (2)
Cl2B	0.0155 (2)	0.0262 (3)	0.0328 (3)	−0.0023 (2)	−0.0045 (2)	−0.0064 (2)
Cl3B	0.0288 (3)	0.0259 (3)	0.0428 (3)	−0.0104 (2)	−0.0143 (2)	−0.0046 (2)
S1B	0.0169 (2)	0.0196 (3)	0.0239 (3)	−0.0055 (2)	−0.0045 (2)	0.0002 (2)
S2B	0.0169 (2)	0.0262 (3)	0.0181 (3)	−0.0022 (2)	−0.0042 (2)	−0.0037 (2)
O1B	0.0285 (8)	0.0374 (9)	0.0212 (8)	−0.0080 (7)	−0.0092 (7)	−0.0065 (7)
N1B	0.0224 (9)	0.0251 (9)	0.0180 (9)	−0.0060 (7)	−0.0013 (7)	−0.0071 (7)
C1B	0.0169 (10)	0.0212 (11)	0.0251 (11)	−0.0058 (8)	−0.0046 (8)	0.0005 (9)
C2B	0.0253 (11)	0.0185 (10)	0.0185 (10)	−0.0073 (9)	−0.0020 (9)	−0.0052 (8)
C3B	0.0332 (13)	0.0463 (15)	0.0239 (12)	−0.0089 (11)	0.0030 (10)	−0.0158 (11)
C4B	0.0149 (10)	0.0209 (10)	0.0196 (10)	−0.0028 (8)	−0.0010 (8)	−0.0053 (8)
C5B	0.0205 (10)	0.0187 (10)	0.0242 (11)	−0.0057 (8)	−0.0015 (8)	−0.0045 (8)
C6B	0.0221 (11)	0.0272 (11)	0.0268 (12)	−0.0113 (9)	−0.0042 (9)	−0.0061 (9)
C7B	0.0163 (10)	0.0279 (11)	0.0270 (12)	−0.0056 (9)	−0.0045 (9)	−0.0005 (9)
C8B	0.0213 (11)	0.0202 (11)	0.0370 (13)	−0.0057 (9)	−0.0047 (10)	0.0017 (10)
C9B	0.0207 (11)	0.0217 (11)	0.0322 (12)	−0.0072 (9)	−0.0018 (9)	−0.0074 (9)

### (3) (N-Methyl-N-phenylcarbamoyl)(trichloromethyl)disulfane Geometric parameters (Å, º)

**Table:**

Cl1A—C1A	1.768 (2)	Cl1B—C1B	1.774 (2)
Cl2A—C1A	1.776 (2)	Cl2B—C1B	1.768 (2)
Cl3A—C1A	1.777 (2)	Cl3B—C1B	1.771 (2)
S1A—C1A	1.824 (2)	S1B—C1B	1.822 (2)
S1A—S2A	2.0202 (7)	S1B—S2B	2.0160 (7)
S2A—C2A	1.856 (2)	S2B—C2B	1.842 (2)
O1A—C2A	1.208 (2)	O1B—C2B	1.211 (2)
N1A—C2A	1.345 (3)	N1B—C2B	1.346 (3)
N1A—C4A	1.440 (3)	N1B—C4B	1.447 (3)
N1A—C3A	1.467 (3)	N1B—C3B	1.460 (3)
C3A—H3AA	0.9800	C3B—H3BA	0.9800
C3A—H3AB	0.9800	C3B—H3BB	0.9800
C3A—H3AC	0.9800	C3B—H3BC	0.9800
C4A—C9A	1.387 (3)	C4B—C9B	1.384 (3)
C4A—C5A	1.389 (3)	C4B—C5B	1.386 (3)
C5A—C6A	1.385 (3)	C5B—C6B	1.385 (3)
C5A—H5AA	0.9500	C5B—H5BA	0.9500
C6A—C7A	1.376 (3)	C6B—C7B	1.385 (3)
C6A—H6AA	0.9500	C6B—H6BA	0.9500
C7A—C8A	1.383 (3)	C7B—C8B	1.387 (3)
C7A—H7AA	0.9500	C7B—H7BA	0.9500
C8A—C9A	1.393 (3)	C8B—C9B	1.387 (3)
C8A—H8AA	0.9500	C8B—H8BA	0.9500
C9A—H9AA	0.9500	C9B—H9BA	0.9500
			
C1A—S1A—S2A	102.38 (7)	C1B—S1B—S2B	104.40 (7)
C2A—S2A—S1A	99.96 (7)	C2B—S2B—S1B	101.59 (7)
C2A—N1A—C4A	123.13 (17)	C2B—N1B—C4B	122.00 (17)
C2A—N1A—C3A	118.95 (18)	C2B—N1B—C3B	119.49 (18)
C4A—N1A—C3A	117.85 (17)	C4B—N1B—C3B	118.00 (17)
Cl1A—C1A—Cl2A	110.05 (11)	Cl2B—C1B—Cl3B	109.89 (11)
Cl1A—C1A—Cl3A	108.33 (11)	Cl2B—C1B—Cl1B	110.25 (11)
Cl2A—C1A—Cl3A	108.62 (11)	Cl3B—C1B—Cl1B	107.66 (11)
Cl1A—C1A—S1A	112.54 (11)	Cl2B—C1B—S1B	103.07 (10)
Cl2A—C1A—S1A	104.78 (11)	Cl3B—C1B—S1B	113.09 (11)
Cl3A—C1A—S1A	112.43 (11)	Cl1B—C1B—S1B	112.83 (11)
O1A—C2A—N1A	125.9 (2)	O1B—C2B—N1B	126.4 (2)
O1A—C2A—S2A	122.09 (16)	O1B—C2B—S2B	122.96 (16)
N1A—C2A—S2A	111.99 (15)	N1B—C2B—S2B	110.65 (14)
N1A—C3A—H3AA	109.5	N1B—C3B—H3BA	109.5
N1A—C3A—H3AB	109.5	N1B—C3B—H3BB	109.5
H3AA—C3A—H3AB	109.5	H3BA—C3B—H3BB	109.5
N1A—C3A—H3AC	109.5	N1B—C3B—H3BC	109.5
H3AA—C3A—H3AC	109.5	H3BA—C3B—H3BC	109.5
H3AB—C3A—H3AC	109.5	H3BB—C3B—H3BC	109.5
C9A—C4A—C5A	120.4 (2)	C9B—C4B—C5B	120.95 (19)
C9A—C4A—N1A	120.05 (19)	C9B—C4B—N1B	118.54 (18)
C5A—C4A—N1A	119.51 (19)	C5B—C4B—N1B	120.51 (18)
C6A—C5A—C4A	119.8 (2)	C6B—C5B—C4B	119.38 (19)
C6A—C5A—H5AA	120.1	C6B—C5B—H5BA	120.3
C4A—C5A—H5AA	120.1	C4B—C5B—H5BA	120.3
C7A—C6A—C5A	120.0 (2)	C7B—C6B—C5B	120.23 (19)
C7A—C6A—H6AA	120.0	C7B—C6B—H6BA	119.9
C5A—C6A—H6AA	120.0	C5B—C6B—H6BA	119.9
C6A—C7A—C8A	120.4 (2)	C6B—C7B—C8B	119.9 (2)
C6A—C7A—H7AA	119.8	C6B—C7B—H7BA	120.1
C8A—C7A—H7AA	119.8	C8B—C7B—H7BA	120.1
C7A—C8A—C9A	120.2 (2)	C9B—C8B—C7B	120.3 (2)
C7A—C8A—H8AA	119.9	C9B—C8B—H8BA	119.8
C9A—C8A—H8AA	119.9	C7B—C8B—H8BA	119.8
C4A—C9A—C8A	119.2 (2)	C4B—C9B—C8B	119.2 (2)
C4A—C9A—H9AA	120.4	C4B—C9B—H9BA	120.4
C8A—C9A—H9AA	120.4	C8B—C9B—H9BA	120.4
			
C1A—S1A—S2A—C2A	92.91 (10)	C1B—S1B—S2B—C2B	−95.23 (10)
S2A—S1A—C1A—Cl1A	−55.40 (11)	S2B—S1B—C1B—Cl2B	−169.19 (7)
S2A—S1A—C1A—Cl2A	−174.96 (8)	S2B—S1B—C1B—Cl3B	−50.59 (12)
S2A—S1A—C1A—Cl3A	67.26 (11)	S2B—S1B—C1B—Cl1B	71.90 (11)
C4A—N1A—C2A—O1A	177.3 (2)	C4B—N1B—C2B—O1B	−172.5 (2)
C3A—N1A—C2A—O1A	0.3 (3)	C3B—N1B—C2B—O1B	−0.8 (3)
C4A—N1A—C2A—S2A	−3.0 (2)	C4B—N1B—C2B—S2B	8.1 (2)
C3A—N1A—C2A—S2A	−179.98 (15)	C3B—N1B—C2B—S2B	179.73 (16)
S1A—S2A—C2A—O1A	10.32 (19)	S1B—S2B—C2B—O1B	6.32 (19)
S1A—S2A—C2A—N1A	−169.40 (14)	S1B—S2B—C2B—N1B	−174.23 (13)
C2A—N1A—C4A—C9A	−72.9 (3)	C2B—N1B—C4B—C9B	93.8 (2)
C3A—N1A—C4A—C9A	104.1 (2)	C3B—N1B—C4B—C9B	−78.0 (3)
C2A—N1A—C4A—C5A	109.7 (2)	C2B—N1B—C4B—C5B	−86.4 (3)
C3A—N1A—C4A—C5A	−73.3 (3)	C3B—N1B—C4B—C5B	101.8 (2)
C9A—C4A—C5A—C6A	0.0 (3)	C9B—C4B—C5B—C6B	−2.1 (3)
N1A—C4A—C5A—C6A	177.36 (19)	N1B—C4B—C5B—C6B	178.12 (19)
C4A—C5A—C6A—C7A	0.8 (3)	C4B—C5B—C6B—C7B	0.7 (3)
C5A—C6A—C7A—C8A	−0.3 (3)	C5B—C6B—C7B—C8B	1.1 (3)
C6A—C7A—C8A—C9A	−1.0 (3)	C6B—C7B—C8B—C9B	−1.6 (3)
C5A—C4A—C9A—C8A	−1.2 (3)	C5B—C4B—C9B—C8B	1.6 (3)
N1A—C4A—C9A—C8A	−178.58 (19)	N1B—C4B—C9B—C8B	−178.57 (19)
C7A—C8A—C9A—C4A	1.7 (3)	C7B—C8B—C9B—C4B	0.2 (3)
